# Catch and relay: brighter near-infrared photoluminescence in lanthanide-based nanoparticles

**DOI:** 10.1038/s41377-026-02384-5

**Published:** 2026-06-22

**Authors:** Liyan Ming, Riccardo Marin

**Affiliations:** 1 Intelligent Optical Nanomaterials (IONs) Lab, Departamento de Quimica Organica, Facultad de Ciencias, Universidad Autónoma de Madrid, Madrid, Spain; 2 Institute for the Advanced Research in Chemical Sciences (IAdChem), Universidad Autónoma de Madrid, Madrid, 28049 Spain

**Keywords:** Nanoparticles, Nanophotonics and plasmonics

## Abstract

A design strategy for NIR-emitting lanthanide nanoparticles resolves the fundamental tension between core-shell passivation and dye sensitization. By engineering a Yb³⁺-doped interlayer as an active energy relay between surface-bound ICG and an heavily Er³⁺-doped core, a ~ 2000-fold brightness enhancement is achieved, enabling high-contrast in vivo vascular imaging.

## Abstract (web summary)

An Yb^3+^-doped shell in lanthanide nanoparticles acts as an active energy relay, funneling the energy absorbed by ICG to Er^3+^ ions in the core, hence boosting NIR-II emission ~2000-fold.

## Graphical abstract

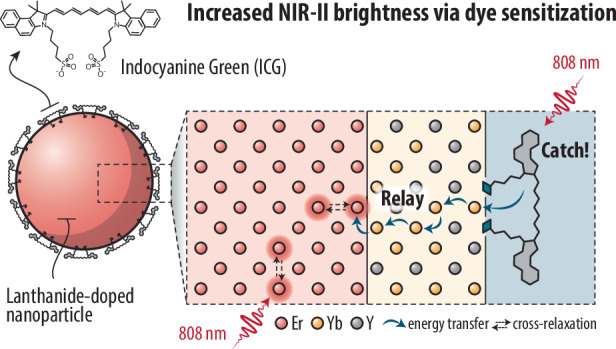

Lanthanide-doped nanoparticles (LnNPs) are among the most used luminescent contrast agents in the biological and preclinical biomedical fields^1,2^. Narrow emission bands, excellent photostability, large pseudo-Stokes shifts, and extended luminescence lifetimes are the differentiators of LnNPs^3,4^. An additional feature of these particles of great appeal in the bio-context is the possibility of tuning their composition and architecture to obtain near-infrared (NIR; 750–2000 nm) emission^5–7^. Indeed, in this wavelength range, tissue-induced photon scattering is reduced and autofluorescence is minimized, thus yielding increased signal-to-background ratios and high-contrast subcutaneous images^8,9^. The combination of NIR-I (750–950 nm) excitation with NIR-II (1000–2000 nm) emission especially represents a practical optimum, balancing deep tissue penetration, minimal water-absorption-induced heating, and accessible laser sources^10^. LnNPs checking all the above boxes have led to incredible strides in the fields of imaging and sensing, thanks to the implementation of advanced techniques such as multiplexing, time-gated imaging, and reliable lifetime-based thermometry^11–13^.

Despite the advantages offered by LnNPs, a major limitation is their reduced brightness compared to, e.g., quantum dots and organic dyes^14,15^. This is a crippling feature in both imaging and sensing, since low signal levels directly translate to poor image quality, reduced temporal resolution, and lowered readout precision^16–18^. Often loosely used to indicate the intensity of the photoluminescence of a species, the term *brightness* is rigorously defined as the product between the two metrics that quantify the efficiencies of photon absorption and photon emission: absorption coefficient (or absorption cross-section) and photoluminescence quantum yield (PLQY), respectively^19,20^. On the one hand, photon absorption by lanthanide ions (Ln^3+^) is intrinsically inefficient, due to the quantum-mechanically forbidden nature of the intraconfigurational 4f-4f electronic transitions that underpin the photophysics of LnNPs^21^. On the other hand, PLQY in LnNPs is often capped by surface and concentration quenching phenomena, which are responsible for non-radiative depopulation of Ln^3+^ emitting states^22^.

Several strategies have been developed to address these issues. Growth of core-shell architectures is often the tool of choice to maximize PLQY, since careful design allows to effectively passivate surface trap states, separate active Ln^3+^ ions from high-vibrational-energy oscillators (e.g., solvent molecules), and compartmentalize different Ln^3+^ ions in selected volumes of the LnNP^23^. This approach has proven highly successful, allowing to achieve percentage values of PLQY in the double digits^24^. Less effective have been the strategies to solve the poor absorption efficiency of LnNPs. Introduction of large amounts of Ln^3+^—i.e., the use of high doping concentrations—enhances the absorption cross-section per LnNP; yet this approach is intrinsically limited by the low absorption cross-section of single Ln^3+^ ions (10^−21^–10^−20^ cm^−2^)^14,25,26^. Another family of approaches make use of a non-Ln^3+^ absorber that more effectively harvest the optical energy of the excitation photons and funnels it to the Ln^3+^ to *sensitize* its emission^16^. Semiconductors^14^, transition metals^27^, and organic dyes^28,29^ are among the most explored species. Dye-sensitization specifically has emerged as a versatile strategy for LnNPs. Initially mostly developed to improve the brightness of upconverting nanoparticles using dyes such as indocyanine green (ICG) and other cyanine derivatives^30–32^, the decoration of LnNPs with dyes has also been extended to NIR-emitting systems to achieve fluorescence-guided tumor resection^33^, in vivo inflammation imaging^34^, and photodynamic therapy^35^. However, growth of core-shell architectures and use of dyes as sensitizers are generally considered strategies at odds. This is because the dye transfers the absorbed energy to the Ln^3+^ ions via Förster resonance energy transfer (FRET): a process that scales as *R*^-6^ (where *R* is the donor-acceptor distance) and generally becomes negligible beyond 10 nm^36^. Thus, the presence of an intermediate shell between the surface-bound donor dyes and the acceptor Ln^3+^ ions frustrates the effort to obtain efficient dye-sensitized photoluminescence.

In their recently published work, Long et al. provide an holistic strategy to enhance the brightness of NIR-emitting LnNPs by simultaneously leveraging careful core-shell design and dye-sensitization, reconceiving the shell as an active *energy relay* component (Fig. 1)^37^. The authors select Er³⁺-based LnNPs as prototypical NIR-II emitters, owing to the ⁴I₁₃_/_₂ → ⁴I₁₅_/_₂ transition at ~1530 nm under 808-nm exctitation^38^. The use of NaErF₄ as core material is ideal in this context since it promotes the effective population of the lowest excited state (⁴I₁₃_/_₂) via cross-relaxation and it maximizes direct Er^3+^ excitation thanks to the high concentration of Er^3+^ ions. The LnNPs design also replaces the conventional inert shell (i.e., devoid of Ln^3+^ ions participating in photon absorption and/or energy transfer processes) with a thin (~2 nm) Yb³⁺-doped interlayer positioned between surface-bound ICG molecules and an Er³⁺-rich NaErF₄ core. This configuration establishes a *cascaded* energy transfer pathway (ICG → Yb³⁺ → Er³⁺), effectively extending the reach of surface excitation into the nanoparticle interior. Systematic comparisons of Er^3+^, Nd^3+^, or Yb^3+^-doped shells reveal competing design principles. An Er³⁺-doped shell shortens the donor-acceptor distance but accelerates energy migration to surface traps, resulting in quenching unless sufficiently thick. Nd³⁺-doped shells are similarly limited by defect coupling. In contrast, Yb³⁺ offers a favorable balance: Its ²F₅/₂ level resonates with the Er³⁺ ⁴I₁₁/₂ state, enabling efficient energy transfer, while its simpler energy-level structure minimizes nonradiative losses. The Yb³⁺ sublattice thus acts as an energy relay, capturing excitation from ICG and redistributing it into the Er³⁺ core, enhancing population of the ⁴I₁₃/₂ emitting state. Time-resolved spectroscopy supports this mechanism. Transient absorption measurements show that the fluorescence lifetime of ICG decreases from 883 to 84 ps upon coupling, corresponding to an energy transfer efficiency of ~90%. Complementary time-resolved luminescence confirms bidirectional energy flow, consistent with a dynamic Yb³⁺-mediated relay. Additional quenching studies indicate that ICG operates primarily via a singlet energy-transfer pathway.

**Fig. 1:**
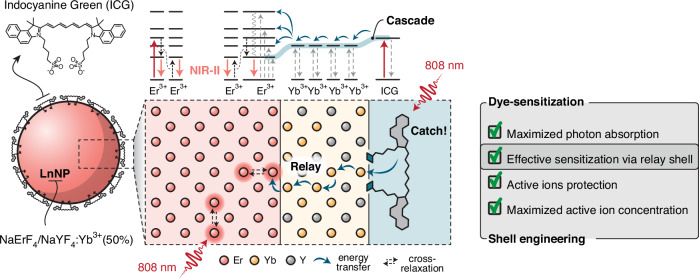
Scheme of the optimized bright, NIR-II emitting LnNP combining dye sensitization and core-shell engineering. Vertical gray dashed lines in the partial energy level scheme indicate electronic transitions through energy transfer processes. Gray wavy lines indicate non-radiative, vibrational de-excitation events

The resulting performance enhancement is substantial. The optimized nanoprobe (NaErF₄/NaYF₄:50%Yb^3+^/ICG) exhibits an ~2000-fold increase in 1525-nm emission relative to the bare core and an ~11-fold improvement over its inert-shell counterpart. After transfer to aqueous media via encapsulation with PEGylated phospholipids, the nanoprobe shows good colloidal stability and enables high-contrast in vivo vascular imaging, resolving vessels of ~220 μm with a signal-to-background ratio of ~3 under 808-nm excitation.

Several directions emerge naturally from this work. The cascaded relay principle is not inherently tied to the Yb³⁺-ICG pairing: Other relay ions with suitable energy-level resonances—or sensitizers with complementary spectral coverage—could be substituted to shift the operating wavelength or excitation band, offering a systematic route for emitters throughout the NIR-II range from a unified design framework. A more immediate question concerns the physical limits of the relay layer itself: Whether there exists an optimal Yb³⁺ concentration and shell thickness at which the trade-off between relay efficiency and Yb³⁺-defect coupling is minimized, and whether this optimum can be predicted from rate-equation modeling rather than determined empirically. An answer to this question would also pave the way for the use of physics-informed machine-learning algorithms to guide the design of optimized dye-Ln^3+^ combinations and LnNP architectures. At the same time, the current reliance on electrostatic ICG adsorption raises practical concerns about dye desorption under physiological conditions—covalent conjugation strategies that preserve coupling geometry while improving stability in vivo would strengthen the translational case considerably. Further modification of the LnNPs surface to introduce active targeting capabilities would broaden the reach of these nanomaterials; yet, it would also require assessment of possible interference of the additional moiety in the sensitization pathway.

Overall, Long et al. establish a clear design principle for overcoming the surface-to-core sensitization mismatch that plagues NIR-emitting dye-sensitized lanthanide probes: Engineer the interlayer not to block energy flow, but to preferentially direct it inwards. Whether this principle proves broadly transferable across lanthanide systems and device contexts will determine its ultimate impact, but it nonetheless represents a substantial conceptual reorientation of the design of NIR-emitting, dye-sensitized LnNPs.
